# The Role of Nuclear Receptors in the Pathogenesis and Treatment of Non-alcoholic Fatty Liver Disease

**DOI:** 10.7150/ijbs.87305

**Published:** 2024-01-01

**Authors:** Zhenhua Yang, Awang Danzeng, Qiumeng Liu, Chenglong Zeng, Lei Xu, Jie Mo, Ciren Pingcuo, Xiaojing Wang, Chao Wang, Bixiang Zhang, Binhao Zhang

**Affiliations:** 1 Hepatic Surgery Center, Tongji Hospital, Tongji Medical College, Huazhong University of Science and Technology, Wuhan 430030, Hubei Province, China.; 2 Clinical Medicine Research Center for Hepatic Surgery of Hubei Province, Wuhan 430030, Hubei Province, China.; 3 Department and Institute of Infectious Disease, Tongji Hospital, Tongji Medical College and State Key Laboratory for Diagnosis and Treatment of Severe Zoonostic Infectious Disease, Huazhong University of Science and Technology, Wuhan 430030, Hubei Province, China.

**Keywords:** NAFLD, NRs, PPARs, FXR, LXR

## Abstract

Non-alcoholic fatty liver disease (NAFLD) is a global health burden closely linked to insulin resistance, obesity, and type 2 diabetes. The complex pathophysiology of NAFLD involves multiple cellular pathways and molecular factors. Nuclear receptors (NRs) have emerged as crucial regulators of lipid metabolism and inflammation in NAFLD, offering potential therapeutic targets for NAFLD. Targeting PPARs and FXRs has shown promise in ameliorating NAFLD symptoms and halting disease progression. However, further investigation is needed to address side effects and personalize therapy approaches. This review summarizes the current understanding of the involvement of NRs in the pathogenesis of NAFLD and explores their therapeutic potential. We discuss the role of several NRs in modulating lipid homeostasis in the liver, including peroxisome proliferator-activated receptors (PPARs), liver X receptors (LXRs), farnesoid X receptors (FXRs), REV-ERB, hepatocyte nuclear factor 4α (HNF4α), constitutive androstane receptor (CAR) and pregnane X receptor (PXR).The expanding knowledge of NRs in NAFLD offers new avenues for targeted therapies, necessitating exploration of novel treatment strategies and optimization of existing approaches to combat this increasingly prevalent disease.

## Introduction

Non-alcoholic fatty liver disease (NAFLD), recently proposed by scholars to be renamed metabolic dysfunction-associated steatotic liver disease (MASLD)^1^, has emerged as one of the most prevalent and widespread liver disorders worldwide^2^, ^3^. Recent epidemiological data have revealed that the incidence of NAFLD has risen to an alarming 32.4% in 2022^4^, surpassing previous estimates and showing a significant increase from a baseline of 25.24% in 2016^5^. Furthermore, NAFLD is projected to become the primary cause of cirrhosis necessitating liver transplantation over the next decade^6^-^8^. Due to the lack of early warning signs, NAFLD can lead to substantial healthcare expenses, economic losses and a significant reduction in health-related quality of life^9^, ^10^. At present, NAFLD lacks an approved therapy, although numerous drugs are progressing in advanced stages of development and researchers remain optimistic about the potential benefits of these therapies^11^-^13^. In light of the soaring incidence of NAFLD, there is an urgent need to accelerate research and development efforts to identify safe and effective therapies for this condition.

**Figure 1:**
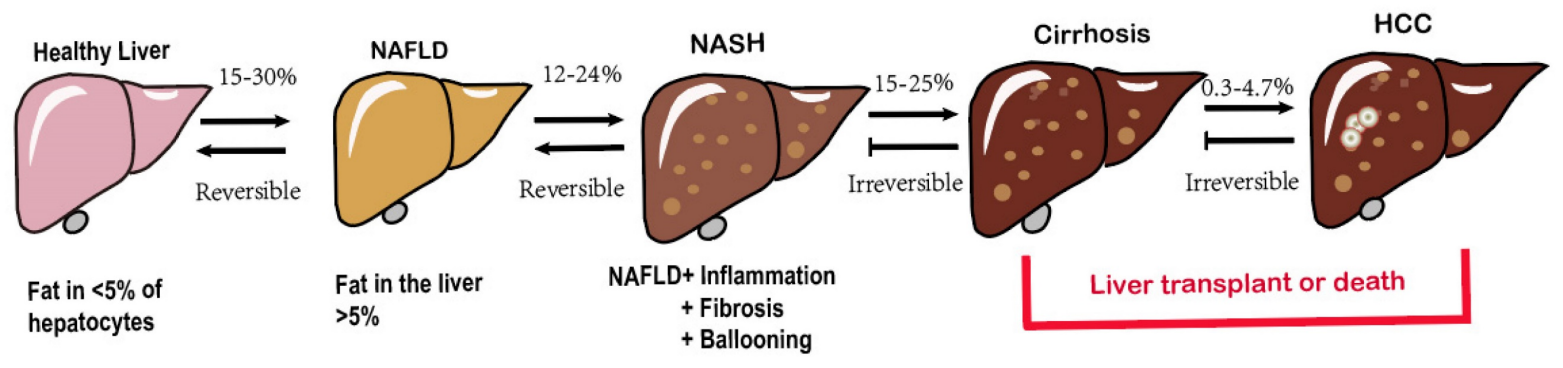
Spectrum of NAFLD.

The hallmark characteristic of NAFLD is macrovescular steatosis, which is defined by the presence of lipid droplets in at least 5% of hepatocytes, without any secondary causes for hepatic fat accumulation, such as clinically significant alcohol consumption^14^. NAFLD, represented the hepatic manifestation of metabolic syndrome, demonstrates a bidirectional association with obesity^15^, T2DM, elevated serum liver enzymes, poor physical condition and hypertension 13, 16-18. Among these factors, T2DM poses a particularly severe threat as it significantly increases the risk of cirrhosis and its associated complications^19^-^21^. Fatty liver disease encompasses a variety of pathological conditions, ranging from lipid accumulation in liver cells (steatosis) to the development of superimposed inflammation (steatohepatitis), characterized by necrotizing inflammation and faster fibrosis progression compared to nonalcoholic liver disease^22^, ultimately culminating in cirrhosis^23^, even hepatocellular carcinoma(HCC) (Fig. 1) 24, 25. The initiation and development of NAFLD are subject to an assortment of factors. Obesity and T2DM closely contribute to the increasing incidence of NAFLD and are directly linked to it^26^. Genetic makeup also has a significant impact on how diseases develop^27^. Additionally, complex interactions between environmental and genetic factors, particularly dietary factors, affect the progression of NAFLD^28^, ^29^. More recently, the gut microbiota has emerged as a significant player in the pathogenesis of NAFLD^30^, ^31^. The mechanisms underlying the development of NAFLD and its complications are intricate and not fully understood^32^. As mentioned earlier, numerous factors work together or synergistically contribute to the onset and progression of NAFLD to non-alcoholic steatohepatitis (NASH) giving rise to the multiple parallel hit hypothesis regarding NAFLD advancement^33^, ^34^.

**Figure 2:**
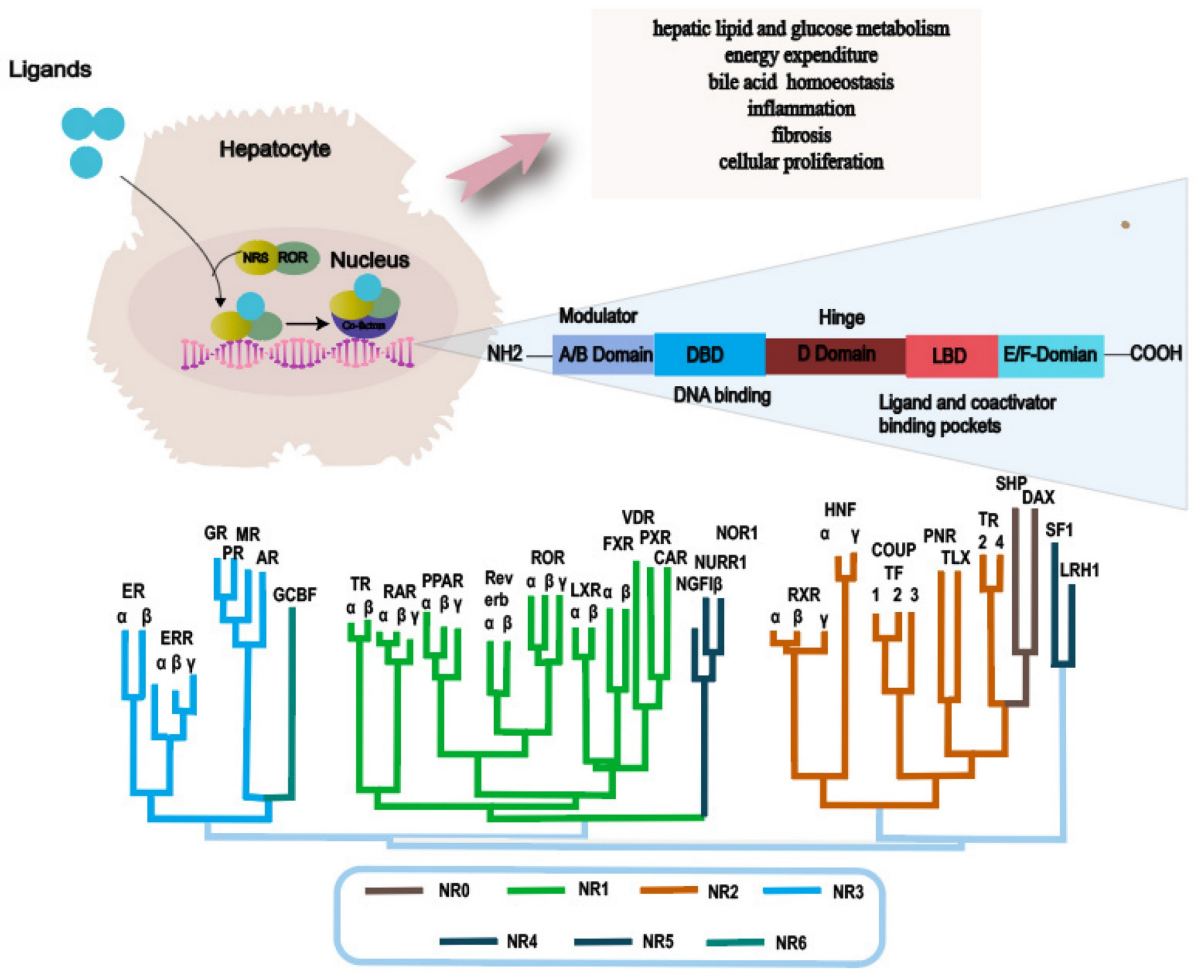
** Main functions of nuclear receptor related to NAFLD and NR phylogenetic tree**. Abbreviations: NAFLD: Non-alcoholic fatty liver disease; NASH: Non-alcoholic steatohepatitis; ER: Estrogen Receptor; ERR: Estrogen-Related Receptor; GR: Glucocorticoid Receptor PR: Progesterone Receptor; MR: Mineralocorticoid Receptor; AR: Androgen Receptor; GCBF: Glucocorticoid-Induced TNFR-Related Protein; TR: Thyroid Hormone Receptor; RAR: Retinoic Acid Receptor; PPAR: Peroxisome Proliferator-Activated Receptor; ROR: Retinoic Acid Receptor-Related Orphan Receptor; LXR: Liver X Receptor; FXR: Farnesoid X Receptor; VDR: Vitamin D Receptor; PXR: Pregnane X Receptor; CAR: Constitutive Androstane Receptor; NGFIβ: Nerve Growth Factor-Inducible Protein β; NURR: Nuclear Receptor Subfamily 4 Group A; NOR1: Neuron-Derived Orphan Receptor 1; RXR: Retinoid X Receptor; HNF: Hepatocyte Nuclear Factor; COUPTF: Chicken Ovalbumin Upstream Promoter-Transcription Factor; PNR: Photoreceptor-Specific Nuclear Receptor; TLX: Tailless Homolog; TR: Thyroid Receptor; SHP: Small Heterodimer Partner; DAX: Dosage-Sensitive Sex Reversal, Adrenal Hypoplasia Critical Region, on Chromosome X; SF1: Steroidogenic Factor 1; LRH1: Liver Receptor Homolog 1.

NRs are a superfamily of transcription factors that are regulated by a myriad of ligands and play an essential role in various physiological processes such as metabolism, immunity and development^35^. The human genome encodes 48 NRs that have been classified into 7 subfamilies, designated as NR0-NR6^36^, ^37^. A typical nuclear receptor consists of five structurally ordered regions for functionality: a variable N-terminal region that is commonly known to possess hormone-independent transactivation function, a conserved DNA binding domain that features two zinc-finger structures, a variable short hinge region that acts as a pivot for flexibility, a conserved ligand-binding domain that modulates interactions between the receptor and ligand, and a variable C-terminal region that contributes to the receptor's stability and specificity^38^-^40^ (Fig. 2).

NRs are mostly activated by chemically diverse small lipophilic ligands, several endogenous and exogenous lipids, such as steroids, retinoids, and phospholipids^41^-^43^. Upon ligands banding, NRs translocate to the nucleus and undergo conformational changes. Subsequently, by binding to reactive elements in the target gene promoter region, it recruits co-regulatory factors to activate or repress target gene expression. Through this process, transcription factors regulate gene expression in response to hormonal and environmental signal^40^. NRs transcriptionally control critical metabolic processes, including liver lipid and glucose metabolism, energy consumption, bile acid (BA) homeostasis, inflammation, fibrosis, and cell proliferation^44^, ^45^. Disruption of these processes contributes to the development and progression of fatty liver disease through the gut-liver-adipose axis and inflammatory signaling pathways^46^. Consequently, NRs have remained prominent drug targets in the forefront of novel therapeutic strategies for NAFLD^47^.

This review focuses on the advancement of the metabolism and agonists of NRs closely related to NAFLD, including PPARs, LXRs, FXRs, REV-ERB, HNF4α, CAR and PXR nuclear receptors. We provide an overview of their impact on the progress of NAFLD, highlighting the interconnected nature of these receptors and the signaling pathways they regulate. Furthermore, we have elucidated the current state of preclinical and clinical studies investigating the efficacy of pharmacological agents targeting NRs in treating NAFLD.

## General characteristics and three subtypes of PPAR

**Figure 3:**
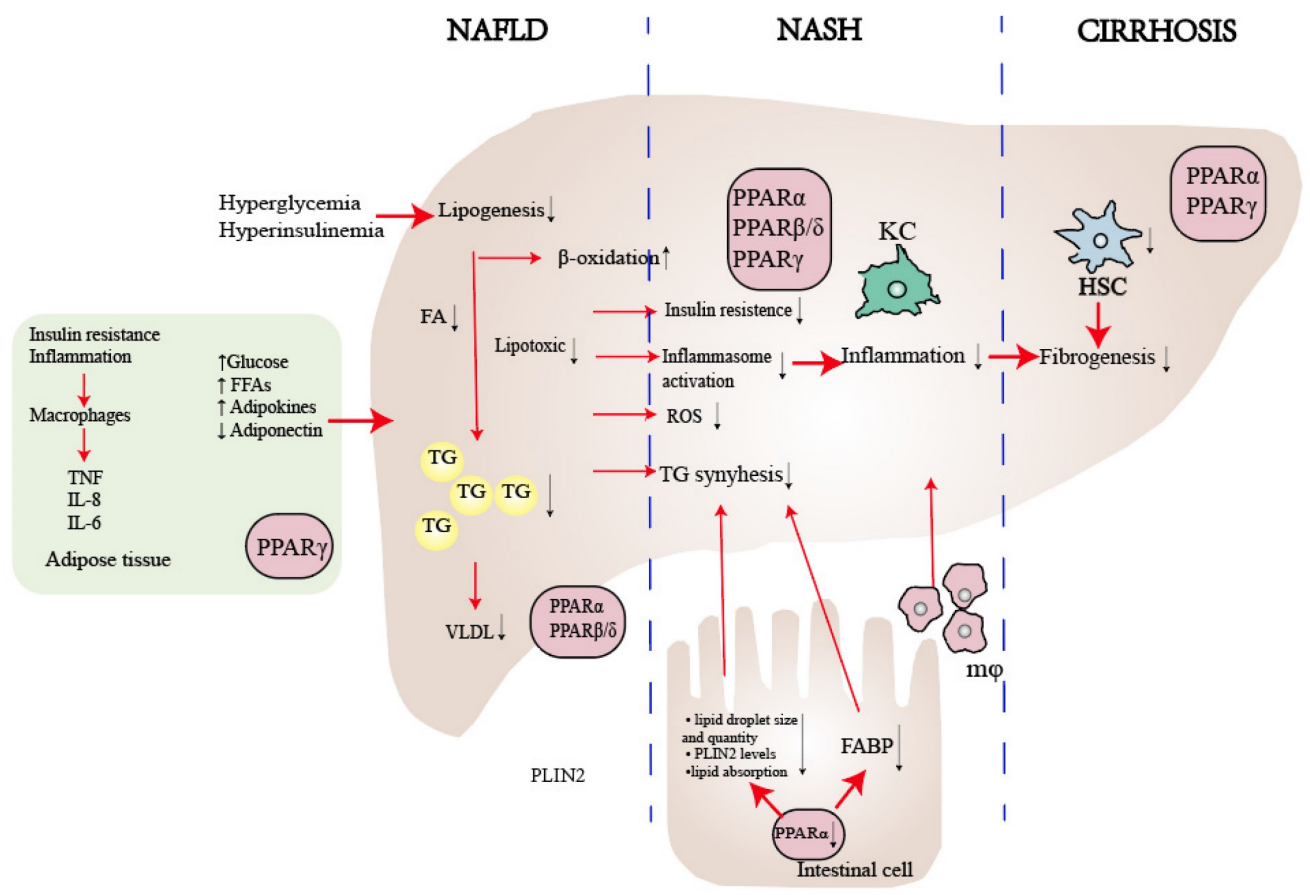
** The role of PPARs in NAFLD.** PPARα plays a crucial role in enhancing lipid metabolism by regulating the flow of lipids, controlling fatty acid transportation, and promoting β-oxidation. Moreover, it contributes to reducing inflammation by influencing liver cells, reducing visceral inflammation, and regulating intestinal permeability. On the other hand, PPARβ/δ suppresses the inflammatory phenotype in macrophages and facilitates the selective activation of a desired phenotype. As for PPARγ, it primarily regulates insulin sensitivity within adipose tissue and serves as the key regulator of hepatic stellate cell (HSC) fate. By preventing HSC activation, PPARγ plays a critical role in inhibiting fibrogenesis. Abbreviations: FFA: Free Fatty Acid; FA: Fatty Acid; TG: Triglyceride; ROS: Reactive Oxygen Species; KC: Kupffer Cell.

The superfamily of ligand-activated transcription factors is known as steroid hormone receptors, which causes the proliferation of peroxisomes^48^. Later on, as key integrators of inflammatory and metabolic signaling^49^, PPARs are the most extensively researched NRs associated with NAFLD^50^. The three PPAR isoforms (PPARα, PPARβ/δ, and PPARγ) exhibit different tissue distributions and play distinct roles in energy metabolism^51^, ^52^. PPARα is extensively expressed in liver, skeletal muscle, brown fat and cardiac tissue, and it regulates energy homeostasis^53^. PPARβ/δ is ubiquitously expressed and enhances fatty acid metabolism^54^. PPARγ is predominant in adipose tissue and causes insulin sensitization and enhances glucose metabolism^55^ (Table 1). The majority of PPARs create heterodimers with retinoic X receptors (RXRs). When a ligand binds, they then attach to peroxisome proliferator response elements (PPREs) in the promoters of target genes, depending on whether co-repressors or co-activators are present^54^, ^56^, ^57^. PPARs play a crucial role in lipid and glucose metabolism, as well as the regulation of energy balance, inflammation, and fibrosis (Fig. 3). Consequently, PPARs represent promising therapeutic targets for a more integrated and coordinated approach to NAFLD treatment^58^-^60^.

## PPARα

PPARα, a nutrient sensor^61^, plays a central role in metabolism and is expressed in tissues with high oxidative activity, such as the liver, skeletal muscle, brown fat, and cardiac tissue^62^. The fasting state stimulates the expression and activity of PPARα. In nocturnal rodents, the peak activity of liver PPARα occurs during the early night^63^, and mice lacking PPARα can develop steatosis under during the fasting phase^64^. In NAFLD, hepatic PPARα expression is initially low but increases alongside histologic improvements following diet/exercise therapy^65^. A recent study found that eight weeks of aerobic training can reduce liver steatosis and inflammation by upregulating the AMP-activated protein kinase (AMPK)-PPARα pathway in the liver^66^, ^67^. In lipid metabolism, PPARα decreases liver fat synthesis through fatty acid transport, apolipoproteins production, genes expressed in β-oxidation 51, 68 or indirectly harmonizing via the LXR signaling pathway^68^, ^69^. In summary, the PPARα-mediated effect on lipid metabolism results in increased levels of serum high-density lipoprotein (HDL) and decreased levels of triglyceride-rich, lipoproteins and triglyceride accumulation in the liver^70^. In carbohydrate metabolism, PPARα regulates the expression of genes involved in gluconeogenesis, the process by which the liver produces glucose from non-carbohydrate sources^71^. PPARα also regulates the expression of genes involved glycogen breakdown and glucose release into the bloodstream 72. Recently, gut-liver crosstalk has been recognized as playing a crucial role in regulating the progression of NAFLD^31^, ^73^, ^74^. A novel study demonstrates that the intestinal PPARα- Fatty acid binding protein 1(FABP1) axis is involved in controlling dietary fatty acid uptake, which in turn modulates obesity and NAFLD 75. Another study has shown that deletion of intestinal PPARα leads to a reduction in both the size and quantity of lipid droplets, decreased fatty acid transport, and depletion of perilipin 2 (PLIN2), a critical regulator of lipid droplet formation in systemic lipid metabolism (Fig. 3)^76^.

### Current clinical trials targeting PPARα signaling for treatment

Both animal and in vivo models have shown the potential therapeutic benefits of PPAR agonists for NAFLD. Recent research has shown that fenofibrate, a PPARα agonist, reduces lipid levels in an mTOR-independent manner by activating autophagy and transcription factors E3 (TFE3) and EB (TFEB), which subsequently decreases hepatic fat accumulation^77^. Unfortunately, fenofibrate has minimal effect on insulin sensitivity or liver histology[78, 79]but exhibits better therapeutic advantages when combined with a PPARγ agonists rather than used alone^80^. Pemafibrate, a new and specific modulator of PPARα, has demonstrated advantageous effects on liver histology and liver enzymes in both preclinical NAFLD models and in individuals with diabetes and dyslipidemia^81^. In a double-blind, placebo-controlled, randomized multicenter phase Ⅱ trial, Pemafibrate did not decrease liver fat content but significantly reduced MRE-based liver stiffness^82^. Furthermore, it is important to note that species differences exist in cellular responses following PPARα activation between mice and humans^83^. This discrepancy could partially clarify why the effectiveness of isolated PPARα agonism shown in preclinical data has not been achieved in histological improvements in NAFLD patients^59^.

## PPARβ/δ

PPARβ/δ is also known to play a critical role in liver metabolism^57^. It is primarily expressed in hepatocytes, Kupffer cells, sinusoidal endothelial cells and hepatic stellate cells (HSCs) 84. Nevertheless, it has been reported that the transcription and activation of PPARβ/δ are decreased in the livers of NAFLD patients in comparison to that of healthy individuals^85^. In lipid metabolism, activation of PPARβ/δ in the liver of mice can suppress the expression of sterol regulatory element binding protein-1c (SREBP-1c), which in turn reduces liver steatosis^86^. PPARβ/δ also inhibits hepatic steatosis and slows down NAFLD progression by regulating the very low-density lipoprotein receptor (VLDLR)^87^. Interestingly, the functions of PPARα and PPARβ/δ in the liver appear to be similar, implying that PPARβ/δ is the primary regulatory factor in liver intermediate metabolism^88^. However, PPARβ/δ cannot compensate for PPARα in PPARα deficient mice^64^. In addition, studies have shown that PPARβ/δ prevent fat toxicity by reducing levels of saturated fatty acids^89^, ^90^. Apart from its role in liver metabolism, PPARβ/δ also plays an important part in modulating inflammation. Ligands binding to PPARβ/δ are associated with the induction of anti-inflammatory signals and phenotypes in Kupffer cell^88^, although the mechanism of its anti-inflammation role is not yet fully understood.

In conclusion, activation of PPARβ/δ results in decreased metabolic disturbances and insulin resistance in the liver, as well as the alternative activation of Kupffer cells with anti-inflammatory properties^91^. The selective PPARβ/δ agonist Seladelpar has shown improvements in insulin sensitivity and steatohepatitis in NASH patients^92^. However, CymaBay Therapeutics discontinued clinical trials of Seladelpar due to alarming results such as portal inflammation, along with plasma cells, interface hepatitis, and localized bile duct irregularities in initial end-of-treatment liver biopsies of NASH patients^93^.

## PPARγ

PPARγ performs specific functions in various tissues and cell type, with its primary role being in adipose tissue^94^. In individuals with NAFLD, the expression levels of PPARγ in the liver are significantly elevated^95^. PPARγ regulates a variety of target genes in adipocytes that are responsible for lipid uptake and storage, the production of inflammatory cytokines, and the secretion of adipokines that enhance insulin sensitivity^96^. In the liver, PPARγ stimulates the uptake of free fatty acids through FABP4-mediated fatty acid synthase (FASN) expression to increase triglyceride levels in hepatocytes. PPARγ also enhances the transcription of SREBP-1c, which in turn activates additional adipogenic genes and leads to the conversion of pyruvate into fatty acids^97^, ^98^ In addition, PPARγ regulates various processes in hepatocytes, kupffer cells and HSCs, and the latter two play a pivotal role in the progression of hepatic inflammation, the development of fibrosis, and ultimately, patient outcome^99^.

Interestingly, compared to traditional fatty liver models such as HFD feeding, chronic alcohol feeding, and leptin gene deletion, Gao et al. have developed a mouse model of acute steatohepatitis (HFD-plus-binge ethanol model) and successfully demonstrated hepatic PPARγ was found to induce steatosis through the upregulation of fat-specific protein 27 (Fsp27) and concurrently mitigate neutrophil infiltration by suppressing CXCL1, a chemokine involved in neutrophil recruitment^100^.

### Current clinical trials targeting PPARγ signaling for treatment

Approaches that reduce fat mass or shrink enlarged adipocytes (like weight loss) or improve insulin sensitivity in adipose tissue through medication (such as thiazolidinediones (TZDs)) are successful in treating NAFLD^101^, ^102^. This success is attributed to the reciprocal communication between the liver and adipose tissue, which adjusts to changes in energy requirements^59^.A recently study found that GW9662, a PPARγ antagonist, attenuates NAFLD progression by reducing hepatic steatosis, inflammation, and oxidative stress^103^. In phase Ⅱb trials, pioglitazone notably improve liver histology liver histology features such as steatosis, inflammation, and ballooning, but not other histological features of NASH^7^, ^104^, ^105^. However, pioglitazone is particularly effective in patients with NASH and prediabetes or T2MD^101^, ^106^. The negative side effects associated with PPARγ activation, including weight gain, fluid retention, increased risk of cardiovascular incidents, and bone fractures, limit the widespread use of pioglitazone in treating NASH^107^. The recently created dual agonists for PPARα/γ, G4 and G5, reduce known side effects while improving systemic glucose metabolism, hyperlipidemia, and markers of liver injury in rats with insulin resistance induced by a high-fructose diet^108^. Saroglitazar, a different dual PPARα/γ agonist, improved steatosis, lobular inflammation, hepatocellular ballooning, and fibrosis levels in an animal model of NASH 109 and it was found to ameliorate ALT, liver fat content, insulin resistance, and atherogenic dyslipidemia in NASH patients, along with positive histological indications^110^. MSDC-0602K was intentionally engineered to diminish direct binding to PPARγ^111^, yet it maintains its ability to inhibit the mitochondrial pyruvate carrier (MPC), which likely contributes to its beneficial effects on energy metabolism and glucose uptake 112. Phase II clinical trials of the MSDC-0602K drug have shown promising results in obese individuals, including reduced glucose and insulin levels, as well as improved liver steatosis, without any adverse side effects^113^, ^114^. Preliminary data also indicates that MSDC-0602 might not possess the risk of bone loss associated with direct PPARγ agonists^115^, ^116^. Comparing with effects of the single or dual PPAR agonists, Lanifibranor, PPAR pan-agonist, improves all histological features of steatohepatitis in mice model of NASH, including liver fibrosis^60^, ^117^, ^118^. Likewise, in a phase Ⅱ clinical trial of Lanifibranor, there is evidence supporting its potential to provide benefits regarding numerous secondary endpoints, including hepatic fibrosis, lipid profile, and glycemic control^119^.

## LXR

LXR, the potential glucose sensor^120^, comprises two isotypes, LXRα and LXRβ. It functions as nuclear receptors with crucial roles in lipid metabolism^69^, ^121^, ^122^, regulating immunity^123^, and exhibiting anti-inflammatory activity^124^. LXRα is predominantly found in metabolically active tissues and cells such as the liver, intestine, adipose tissue, and macrophages, while LXRβ is more universally expressed^125^-^127^.

In hepatic metabolism, LXR serves a dual role. On the one hand is LXR directly increases SREBP-1c, FASN, stearoyl-coenzyme A desaturase 1 (SCD-1) and acetyl-CoA carboxylase (ACC), resulting in detrimental liver lipid deposition and hypertriglyceridemia^128^. An increase in LXR expression has been demonstrated to correlate with the worsening of NASH^127^, ^129^. Thus SR9238, an LXR inverse agonist, decreases the expression of genes encoding DNL enzymes, hepatic steatosis^130^, and plasma liver enzymes in NAFLD mice model. It is worth noting that SR9238 treatment significantly suppressed hepatic inflammation and decreased hepatic fibrosis 127, 131. On the other hand, LXRs regulate reverse cholesterol transport (RCT), which eliminates excess cholesterol through bile and feces after reaching the liver^126^. This process is facilitated by ATP Binding Cassette Subfamily A Member 1(ABCA1) and ATP binding cassette subfamily G member 1(ABCG1) in macrophages, both of which are directly targeted by LXR 132, 133. Intriguingly, pharmacological activation of LXR increases cholesterol removal through feces, regulates cholesterol balance, produces anti-inflammatory effects, and improves insulin sensitivity by upregulating ABCG5/G8^69^, ^134^. However, the opposing pharmacodynamic effects of LXR in the treatment of NAFLD make it difficult to develop targeted drugs^127^.

### Current clinical trials targeting LXR signaling for treatment

A recent study has shown that inhibiting phosphorylation at Ser196 in LXRa can retard the progression of NAFLD in mice that are fed a high-fat and high-cholesterol (HFHC) diet^135^. Additionally, intranuclear MiR-552-3p has been found to suppress metabolic gene expression in vitro and exhibit positive impacts on glycolipid metabolism in vivo by modulating LXRα^136^. Although various selective LXR agonists, such as desmosterol, GW6340, and the LXRβ agonist LXR-623, have shown good tolerability, they are less commonly used for NAFLD treatment^137^, ^138^. Further data is necessary to assess the safety and effectiveness of LXR agonists in NASH therapy.

## FXR

FXR, the primary controller of BA synthesis^139^, is predominantly expressed in the liver and intestines, with lower levels found in the kidneys, adipose tissue, and adrenal glands^140^, ^141^. Chenodeoxycholic acid (CDCA) and cholic acid (CA) serve as endogenous ligands for FXR^142^. Recent studies have revealed that FXR acts as an enterohepatic regulator, controlling BA balance, lipid and glucose metabolism, and inflammation^143^-^146^. BA synthesis, which accounts for approximately 90% of daily cholesterol output in the body^147^, has gained growing interest as a predictive indicator in NASH due to the increased BA levels observed in this condition^148^, ^149^. In a negative feedback loop controls, FXR is the pathway connecting the liver and intestine and regulates BA synthesis through two main pathway: the hepatic FXR-small heterodimer partner (SHP) pathway and the intestinal FXR- FGF15/19 pathway(FGF15 in mice^150^; FGF19 in human)^151^, ^152^. On the one hand, activation of hepatic FXR through BAs mediates induction of SHP expression, which belongs to the atypical nuclear receptor family and promotes inhibition of SREBP1c, thus leading to reduced hepatic synthesis of triglycerides 144. FXR can limit the build-up of fat in the liver by enhancing fatty acid oxidation through the activation of hepatic PPARα and by clearing plasma VLDL triglycerides 151, 153, 154. On the other hand, upon FXR activation in the ileum, human FGF19 is secreted into the bloodstream. It exerts its inhibitory action on BA synthesis and gluconeogenesis by activation two different mechanisms. Firstly, intestinal FXR agonists decrease intestinal lipid absorption by relying on BAs. Secondly, they selectively reduce the synthesis of monounsaturated fatty acids in the liver by inhibiting the expression of three key lipid synthesis genes: Scd1, Lpin1, and Dgat2, however, they do not affect Shp and Srebp-1c^155^. Mice lacking FXR exhibit notably elevated levels of serum and hepatic triglyceride, cholesterol, and free FA levels 156. The removal of serum lipoproteins is contingent upon the function of FXR and is a FGF receptor 4 (FGFR4) located on the hepatocyte surface^157^, ^158^, ultimately reducing liver steatosis and insulin resistance^159^ (Fig. 2). However, the FGF19 agonists are not recommended due to their association with HCC^160^. Clifford et al. have shown that FXR activation reduces liver triglycerides through mediated through the regulation of gene expression of several key targets, including VLDLR, scavenger receptor B1, Syndecan-1, ApoCII/III and FA translocase (FAT/CD36)^161^.

### Current clinical trials targeting FXR signaling for treatment

FXR has become a prominent area of research for NAFLD treatment, with studies exploring the potential benefits of FXR agonists on NAFLD in both animal models and in vivo^148^, ^153^, ^162^. Obese mice treated with the gut-specific FXR agonist, Feraxamine, demonstrated improvements in obesity, insulin resistance, and steatosis^163^. Similarly, another FXR agonist, WAY-362450, reduced liver fibrogenesis and inflammation without triglyceride enrichment in methionine-choline deficient (MCD) diet-fed mice^164^. The non-BA FXR agonist, Tropifexor, has shown favorable outcomes in various preclinical NASH models^165^, ^166^. Furthermore, a phase Ⅱ clinical study has revealed that Cilofexor, a small molecule FXR agonist, has the potential to decrease fat accumulation and fibrosis in patients with NASH^167^, ^168^. Obeticholic acid (OCA) is a potent and specific FXR agonist^169^, ^170^. Key studies examining OCA include the FLINT trial[171]and the REGENERATE trial^172^, ^173^. OCA is the first FXR agonist to complete phase Ⅲ clinical trials in NASH patients, although the primary goal of NASH resolution was not achieved^170^, ^174^.Consequently, the FDA has still not approved OCA for NASH, owing to consistently increased pruritus^173^ and levels of serum LDL and decreased levels of serum HDL^175^ (Table 2).

## REV-ERB and HNF4α

REV-ERB exists two isotypes, REV-ERBα (NR1D1) and REV-ERBβ (NR1D2), and it regulates central and peripheral circadian clocks, lipid and glucose metabolism^176^, and inflammation in the development of NAFLD^177^, ^178^. Treatment with a REV-ERB agonist improves hepatic health by reducing fat mass, improving dyslipidaemia and hyperglycaemia and suppressing hepatic fibrosis and inflammatory response in diet-induced obese mice^176^, ^178^. Central and peripheral circadian clocks as a crucial role in maintaining metabolic homeostasis in the development of liver diseases 179. In liver circadian rhythms, hepatocytes are the peripheral clock that negatively impacts metabolism and overall health^180^. However, the function of REV-ERB can help restore the synchronization of liver clocks, which are frequently disturbed in conditions such as NAFLD, NASH, and metabolic syndrome^181^. In contrast to other nuclear receptors, REV-ERBs function as transcriptional repressors, attracting corepressors when their natural ligand, the iron-centered porphyrin heme, is present^182^, ^183^. REV-ERB is a recognized inflammatory regulator that directly regulates IL-1β, IL-6, TNFα, and the NOD-like receptor protein 3 (NLRP3) inflammasome^181^. NLRP3 activation occurs in NAFLD and increases liver inflammation and fibrosis in mouse NASH model^184^, ^185^.

HNF4α is found in large quantities in the liver^186^ and has been observed to be notably reduced in both individuals with NAFLD and mouse models of NASH^187^. Multiple pieces of evidence indicate that HNF4α plays a role in the development of NAFLD^188^. Genetic studies have identified single nucleotide polymorphisms (SNPs) in the HNF4α gene associated with an increased risk of developing NAFLD^186^. Overexpression of hepatocyte HNF4α promotes lipolysis, fatty acid oxidation and VLDL secretion^189^ to reduce hepatic triglyceride accumulation^190^, whereas hepatocyte-specific HNF4α-/- mice has opposite effects^186^, ^191^, ^192^. In addition, hepatic HNF4a is markedly repressed in NASH and liver fibrosis^193^, ^194^. The crucial role of HNF4α in regulating hepatic lipid metabolism and maintaining liver homeostasis makes it a promising therapeutic target for NAFLD. HNF4α regulates liver fat storage by inducing lipophagy, a process that breaks down lipid droplets, and this effect can be reversed with a potent HNF4α agonist^195^. AAV8-mediated overexpression of HNF4a has been shown to attenuate HFD-induced NAFLD and NASH^190^. Sirtuin2, an NAD+-dependent deacetylase, largely alleviates insulin resistance, hepatic steatosis, and systematic inflammation in HFD-fed mice by binding to and deacetylating HNF4α^196^. However, further research is needed to fully understand the safety and efficacy of therapies targeting HNF4α in humans.

### CAR and PXR

CAR, a member of the NR1I3 family of nuclear receptors, is almost exclusively expressed in the liver and primarily functions as a xenobiotic nuclear receptor^197^. In comparison to the classical NRS, CAR can directly or indirectly activate ligand binding through nuclear translocation from the cytoplasm^198^. In carbohydrate metabolism, CAR activation decreases glucose production in the liver by suppressing the expression of the crucial gluconeogenic genes PEPCK and G6P^199^. The activation of CAR leads to a reduction in SREBP-1c levels by promoting the expression of insulin-induced gene 1 protein (INSIG-1), which hinders the proteolytic activation of SREBPs 200. Additionally, CAR activation increases the expression of the phase II enzyme SULT2B1b, which leads to a decrease in SREBP-1c expression. SULT2B1b also plays a role in sulfating and inactivating oxysterol agonists for LXR^199^. Peroxisome proliferator-activated receptor-gamma coactivator 1α (PGC-1α) acts as a bridge connecting PPARs and CAR, as a transcriptional coactivator interacting with nuclear receptor PPARs. CAR regulates the degradation of PGC-1α by recruiting E3 ligase targeting PGC1α and promoting its ubiquitination in the liver^201^.

PXR, a member of the NR1I2 family of nuclear receptors, is predominantly expressed in the liver^202^. Initially, PXR was defined as the main regulatory factor for exogenous reactions, similar to CAR, and its function was expanded to include lipid and glucose metabolism during the past years^203^. However, the role of PXR in NAFLD is debated, and both preclinical and clinical studies have yielded controversial results. Activation of PXR transcriptional activity has opposite effects on gluconeogenesis in rodents and humans^204^. Despite the presence of opposing evidence, the preponderance of the available data indicates that activation of PXR in mice subjected to a HFHC diet elicited the characteristic features of NAFLD and NASH, including steatosis, inflammation, and lipotoxicity. Karpale et. al. have proposed that the phenomenon of pseudo-improvement in glucose tolerance, where PXR activation resulted in aggravation of liver steatosis without being reflected in systemic glucose tolerance. The phenomenon is explained by remodulation of glucose metabolism 205.

In conclusion, further research is necessary to elucidate the potential roles of PXR and CAR in the development and progression of NAFLD/NASH, as well as to determine their temporal functions in the various stages of this complex disease.

### Nuclear receptor crosstalk

Current strategies relying solely on a 'single-drug' or 'single-target' approach are inadequate in addressing the challenges posed by nuclear receptor ligands in clinical settings. Various nuclear receptors may exhibit shared sets of target genes, indicating overlapping regulatory functions^206^. An example of this is PPARα, through crosstalk with LXR, indirectly regulates the transcription of SREBP1c. Additionally, LXR and PPARα agonists exhibit synergy with insulin in inducing the expression of lipogenic genes like FAS and acetyl-CoA carboxylase 1 (ACC1)^207^. The crosstalk between PPAR and LXR is known to share a considerable number of target genes as they recognize similar response elements^208^. Alternatively, these receptors can also govern distinct genes associated with a common downstream biological process or pathway 206. In the context of lipid metabolism, hepatic FXR directly downregulate three key lipogenic genes, SCD1, LPIN1 and DGAT2^155^. Whereas, PPARα regulates the expression of lipogenic genes mainly through the ChREBP, SREBP1c and LXR pathways^209^. Understanding the intricacies of nuclear receptor crosstalk is essential for developing targeted therapies that can manipulate these signaling pathways for therapeutic purposes.

## Concluding remarks and future perspectives

NAFLD poses a significant health challenge, and researchers have identified several potential molecular targets for its treatment. These targets primarily involve four pathways: hepatic lipid metabolism, inflammation, intestinal flora, and anti-liver fibrosis. In recent years, significant progress has been made in developing metabolic NR ligands and understanding their role in liver physiological regulation. However, despite these advances, there are still challenges in discovering effective new strategies for treating NAFLD. Further research is needed to unravel the complex relationships between different NRs, their regulatory pathways, and their interactions with other metabolic factors during the progression of NAFLD. Exploring new drugs targeting NRs, such as selective PPAR modulators and FXR agonists, may improve treatment efficacy, minimize side effects, and overcome the limitations of current NAFLD treatment. In addition, the recognition of gut-liver crosstalk's significance in NAFLD progression emphasizes the need to study the role of NRs in gut-liver interactions and develop targeted interventions based on these findings. The combination of different NR-targeted drugs or their integration with lifestyle interventions can provide a more comprehensive and effective approach for managing NAFLD.

## Figures and Tables

**Figure 4:**
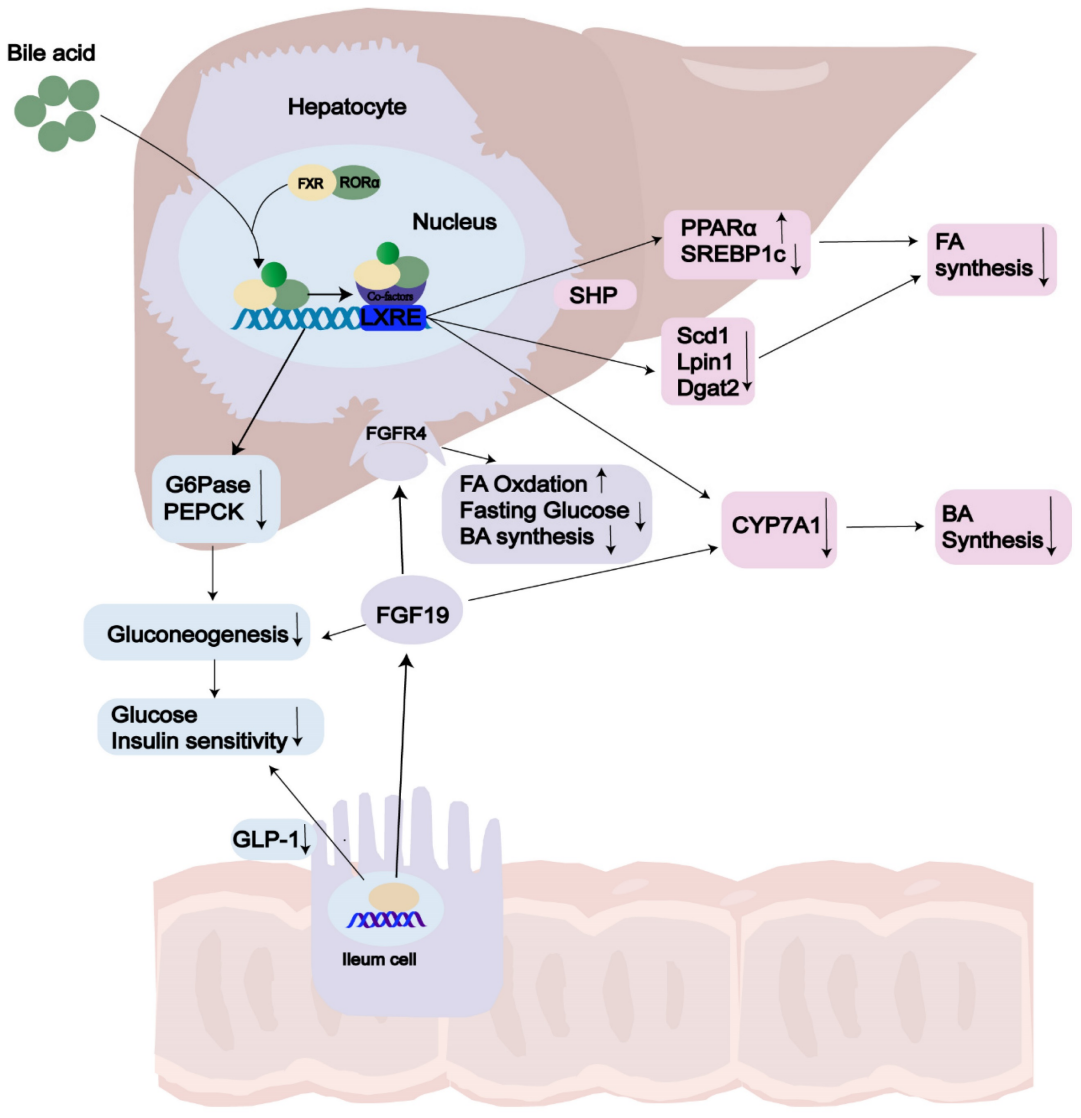
** The role of FXR in NAFLD.** Activation of FXR by bile acid (BA) ligands leads to a reduction in bile acid production and an enhancement of lipid and glucose metabolism. In terms of the bile acid pathway, this activation results in the upregulation of FGF19 and the downregulation of CYP7A1, consequently leading to a decrease in bile acid synthesis. Additionally, the activation of FXR increases the levels of SHP, which subsequently lowers the expression of SREBP1c, PEPCK, and G6Pase. As a result, there is an increase in both lipid and glucose metabolism. Abbreviations: GLP-1: Glucagon-Like Peptide 1; FGF19: Fibroblast Growth Factor 19; FGFR: Fibroblast Growth Factor Receptor; FA: Fatty Acid; BA: Bile Acid; LXRE: Liver X Receptor Response Element; SHP: Small Heterodimer Partner; CYP7A1: Cholesterol 7 Alpha-Hydroxylase.

**Table 1:** The main roles and agonists of PPARs

Isotypes	PPARα		PPARβ	PPARγ	
Tissue expression	Liver Skeletal muscle Brown fat Cardiac tissue		Ubiquitously expressed	Adipose tissue	
Natural ligands	FA Eicosanoids Phospholipids		FA VLDL components	FA Arachidonic acid metabolites	
biolaogical functions related to NAFLD	FA catabolism Ketogenesis FGF21 production Anti-Inflammatory		FA catabolism Lipoprotein metabolism Anti-Inflammatory Glucose utilization	Adipogenesis Adipose FA storage Adipokine secretion Anti-Inflammatory	
Main single agonists	Fenofibrate (NCT02781584)	Pemafibrate (NCT03350165)	Seladelpar	MSDC-0602K (NCT02784444)	Pioglitazone (NCT00063622)
Effect	Plasma triglycerides↓^210^	MRE-based liver stiffness↓	Improves insulin sensitivity and steatohepatitis	Improves liver steatosis	Improve liver histology
Clinical status	Phase Ⅱ	Phase Ⅱ	Pause	Phase Ⅱ	Phase Ⅱ

**Table 2:** Current clinical trials for treatment of targeting to NR signaling

Pharmacologic Compound	Drug Targrt	Effects	Clinical Trials
Saroglitazar^110^, ^211^	PPAR α/γ	Improved glucose/FFA metabolism TG↓, HDL-C↑, neural effect on LDL-C, ALT↓	NCT03061721 PhaseⅡ
Elafibranor	PPAR α/σ	Improved glucose/FFA metabolism	NCT02704403 Phase Ⅲ, trial has been discontinued
Lanifibranor	Pan PPAR	Improved glucose/FFA metabolism TG↓, HDL-C↑, neural effect on LDL-C, ALT↓	NCT03008070 PhaseⅡ, trial has positive results on histology
Cilofexor	FXR	Serum BA↓ Hepatic Steatosis ↓	NCT02854605 PhaseⅡ
Obeticholic acid	FXR	Fibrosis↓ Hepatic inflammation↓	NCT01265498 (FLINT, PhaseⅡ) NCT02548351 (REGENERATE, Phase Ⅲ)

## Abbreviations

NAFLD: non-alcoholic fatty liver disease
NRs: nuclear receptors
PPARs: peroxisome proliferator-activated receptors
LXRs: liver X receptors
FXRs: farnesoid X receptors
HNF4α: hepatocyte nuclear factor 4α
CAR: constitutive androstane receptor
PXR: Pregnane X receptor
MASLD: metabolic dysfunction-associated steatotic liver disease
HCC: hepatocellular carcinoma
NASH: non-alcoholic steatohepatitis
BA: bile acid
RXRs: retinoic X receptors
PPRE: peroxisome proliferator response elements
AMPK: AMP-activated protein kinase
HDL: high-density lipoprotein
FABP1: Fatty acid binding protein1
PLIN2: perilipin 2
TFE3: transcription factors E3
TFEB: transcription factors EB
HSCs: hepatic stellate cells
Fsp27: fat-specific protein 27
SREBP-1c: sterol regulatory element binding protein-1c
VLDLR: very low-density lipoprotein receptor
FASN: fatty acid synthase
TZDs: thiazolidinediones
MPC: mitochondrial pyruvate carrier
SCD-1: stearoyl-coenzyme A desaturase 1
ACC: acetyl-CoA carboxylase
RCT: reverse cholesterol transport
FGF: fibroblast growth factors
ABCA1: ATP Binding Cassette Subfamily A Member 1
ABCG1: ATP binding cassette subfamily G member 1
HFHC: high-fat and high-cholesterol
CDCA: Chenodeoxycholic acid
CA: cholic acid
SNP: small heterodimer partner
FGFR4: fibroblast growth factors receptor 4
MCD: methionine-choline deficient
OCA: Obeticholic acid
NLRP3: NOD-like receptor protein 3
SNPs: single nucleotide polymorphisms
PGC-1α: Peroxisome proliferator-activated receptor-gamma coactivator 1α
ACC1: acetyl-CoA carboxylase 1
